# Switching of biological therapy to dupilumab in comorbid patients with severe asthma and CRSwNP

**DOI:** 10.1007/s00405-024-08461-y

**Published:** 2024-02-12

**Authors:** Cecilia Rosso, Eugenio De Corso, Valerio Conti, Letizia Nitro, Alberto Maria Saibene, Elena Parazzini, Rocco Rinaldo, Sabrina De Pascalis, Flavio Arnone, Stefano Centanni, Claudio Montuori, Leandro Maria D’Auria, Giovanni Felisati, Carlotta Pipolo

**Affiliations:** 1 Otolaryngology Unit, ASST Santi Paolo e Carlo, Department of Health Sciences, Università degli Studi di Milano, Milan, Italy; 2 Unit of Otorhinolaryngology-Head and Neck Surgery, A. Gemelli Hospital Foundation IRCCS, 00168 Rome, Italy; 3 Otolaryngology Unit, ASST Santi Paolo e Carlo, Università degli Studi di Milano, Milan, Italy; 4 Pulmonology Department, ASST Santi Paolo e Carlo, Department of Health Sciences, Università degli Studi di Milano, Milan, Italy; 5 Department of Head-Neck and Sensory Organs, Catholic University of Sacred Heart, 00168 Rome, Italy; 6 Department of Otolaryngology, San Paolo Hospital, University of Milan, via di Rudinì 8, 20154 Milan, Italy

**Keywords:** Nasal polyps, Biological therapy, Monoclonal antibodies, Dupilumab, CRSwNP, Mepolizumab, Omalizumab, Benralizumab

## Abstract

**Purpose:**

Nowadays, several efficacious biologic drugs are used for severe asthma with or without chronic rhinosinusitis with nasal polyps (CRSwNP). However, it has been observed that not all comorbid patients (asthma/CRSwNP) receiving biologic treatment for asthma experience satisfactory control of both conditions equally.

**Methods:**

We selected 20 patients who had both severe asthma and comorbid CRSwNP under biological treatment with benralizumab, omalizumab or mepolizumab with adequate control of asthma but inadequate control of nasal symptoms. Patients were switched to dupilumab and outcomes were evaluated at baseline (T0), at 3 months (T1), at 6 months (T2), at 12 months (T3) and finally at 18 months (T4). Data were collected at each time point including blood tests measuring eosinophil levels and total IgE, SNOT22, ACT, NPS score, rhinomanometry, olfactory testing, and nasal cytology.

**Results:**

The results showed an overall improvement in all the outcomes. Peripheral eosinophilia was observed consistently with existing literature. All patients registered an improvement in sinonasal outcomes, while only one patient had a worsening of asthma. Three patients interrupted the therapy due to various causes: poor asthma control, onset of psoriasis and thrombocytopenia.

**Conclusions:**

The response to a biologic treatment for CRSwNP control may be heterogenous and it seems that patients may benefit from switching improving control in equal measure in the upper and lower airway. Further studies to explore the endotype/phenotype which best fits with each biologic are mandatory to personalize the therapy.

## Introduction

Chronic rhinosinusitis with nasal polyposis (CRSwNP) represents a prevalent condition affecting approximately 1–4% of the general population, with a high impact in the quality of life (QoL) [1]. The dominant inflammation type in diffuse primary CRSwNP in caucasian population appears to be type 2, which manifests itself both at the upper and lower airways following the concept of the "united airways" where paranasal sinuses, bronchi, and lungs are interconnected by the same physiopathology [2, 3]. Accordingly, around 40–60% of individuals with type 2 CRSwNP also have coexisting asthma [4]. In fact, before the advent of dupilumab for isolated CRSwNP, biologics were already used to control severe asthma, showing collaterally a good control of the CRSwNP symptoms in patients having both the diseases [5].

Management of this type of patients is based on a stepwise approach [6, 7]. In cases where both surgery and medical management fail, new biological therapies may be considered [2]. The monoclonal antibodies that have been investigated and approved in many countries for CRSwNP primarily targeting type 2 pathways and namely: interleukin-4 (IL-4)/interleukin-13 (IL-13) path inhibiting the IL-4 receptor alpha (using dupilumab), blocking IL-5 (using mepolizumab or Benralizumab), and neutralizing immunoglobulin E (IgE) through omalizumab [8–13]. Despite the great efficacy of biologic treatments, in subjects presenting both CRSwNP and asthma, not each patient shows improvement and control of both comorbidities in equal measure [14, 15]. Furthermore, it appears necessary in certain circumstances to switching to other biologics due to non-control of CRSwNP or asthma, but still there is poor written in literature about this topic.

This led our multidisciplinary teams to analyze those patients that had undergone a switch from their biologic treatment to dupilumab in those comorbid patients that had not reached control of the CRSwNP with the previous biological therapy started primarily for severe asthma control.

## Materials and methods

Patients who were treated for severe asthma and CRSwNP at ASST Santi Paolo and Carlo, Milan, and at A. Gemelli Hospital Foundation IRCCS Rome and that were switched from a previous biologic to dupilumab; and had at least 1 year of follow-up in our two multidisciplinary outpatient clinics after switching were enrolled in our study. The study was designed as retrospective observational. The study was approved by IRB n° 16775 of Ethical committee. All patients consented to data-collection and use in line with the GDPR in both hospitals. Patients selection was performed from December 2021 to August 2023.

All patients had an established diagnosis of severe persistent asthma (SA) [9] and of CRSwNP as defined in EPOS2020 guidelines [2] All patients had been under treatment with Benralizumab, Omalizumab or Mepolizumab for at least 1-year prior, showing no response in terms of CRSwNP even after previous surgeries and current medical/biologic therapy intended as no improvement in more than one of the following criteria: reduction of NPS score; Improvement of SNOT-22 score; Reduction of OCS (Oral Corticosteroids) consumption; Smell improvement [2]. Switching to dupilumab was chosen by a multidisciplinary team.

Patients presented with a type-2 pattern of inflammation defined according to 2020 EPOS criteria [2]. As parameters may be altered by previous biological therapies which may lower blood levels of IgE and eosinophils, we considered blood tests performed previously to the first monoclonal antibody treatment.

Exclusion criteria were the presence of secondary CRS and the co-presence of systemic diseases which may affect the sinonasal region (e.g. Granulomatosis with Polyangiitis (GPA), eosinophilic granulomatosis with polyangiitis (EGPA)).

Patients were evaluated by our multidisciplinary Unit. They underwent:

- ACT (Asthma Control Test) (score 1–25).
- Total IgE and blood count with differential.
- SNOT 22 (score 0–110).
- Olfactometry (Burghart Sniffin’Stick -Identification test) (score 0–16).
- Rhinomanometry (Rhinolab 4-Phase-Rhinomanometry (4PR)).
- Endoscopy evaluating Nasal Polyp Score (NPS) (score 0–8).

All patients have been evaluated before the switching of therapy to dupilumab (T0), and at 3 months (T1), 6 months (T2), 12 months (T3) and 18 months (T4) after the start of treatment. Demographic data were collected at T0 evaluation regarding age, gender, BMI, type of work, previous surgeries, family history for CRSwNP or asthma, comorbidities such as allergies, ASA intolerance and smoking, type of previous monoclonal antibody, number of OCS cycles during the last year, comorbidities such as allergies, ASA intolerance and smoke.

Informed consent was obtained by all patients.

Evaluation of outcomes from T0 to T4 was carried out in terms of asthma and CRSwNP control. Data were analyzed with SPSS Version 27.0 (IBM Corporation. Armonk, NY. US). Since the Kolmogorov–Smirnov normality test indicated that our data did not follow a normal distribution (*p* < 0.001), we chose to use non-parametric tests: a Wilcoxon signed-rank test was carried out to evaluate significance of main nasal outcomes (SNOT22 and NPS).

This observational study followed the STROBE reporting guidelines.

## Results

In this study we enrolled 20 consecutive patients with diagnosis of severe asthma and CRSwNP treated with Omalizumab/Mepolizumab/Benralizumab, who had non-satisfactory nasal outcomes [10] in terms of sinonasal symptoms and nasal endoscopy and that were followed in our multidisciplinary outpatient clinics. Demographic data are resumed in Table 1. Mean time period of therapy with previous biologic was 15.1 months (SD 3.85).

**Table 1:** Demographic data of patients

Patients’ characteristics	
Age (mean)	54 (SD 8.93)
Smokers (no of patients)	1 (5%)
BMI (mean)	26 (SD 5.85)
Allergic to inhalants (no of patients)	11 (55%)
Family history of allergies (no of patients)	2 (10%)
Widal Syndrome (no of patients)	3 (15%)
Non-controlled asthma (no of patients)	2 (10%)
Non-responders CRSwNP (no of patients)	20 (100%)
Previous surgery (no of patients)	20 (100%)
Number of previous surgeries (mean)	2.02 (SD 3.47)
Months since last surgery (mean)	57.03 (SD 42.81)

Non-controlled asthma is intended as non-complete pharmacological control of asthma under biologic therapy, requiring at least 1 OCS cycle per year. Non-controlled CRSwNP is intended as CRSwNP requiring at least 2 cycles of OCS per year. Previous surgery is intended as a number of patients who underwent at least one surgical procedure before starting biologic therapy

*SD* standard deviation

**Fig. 1:**
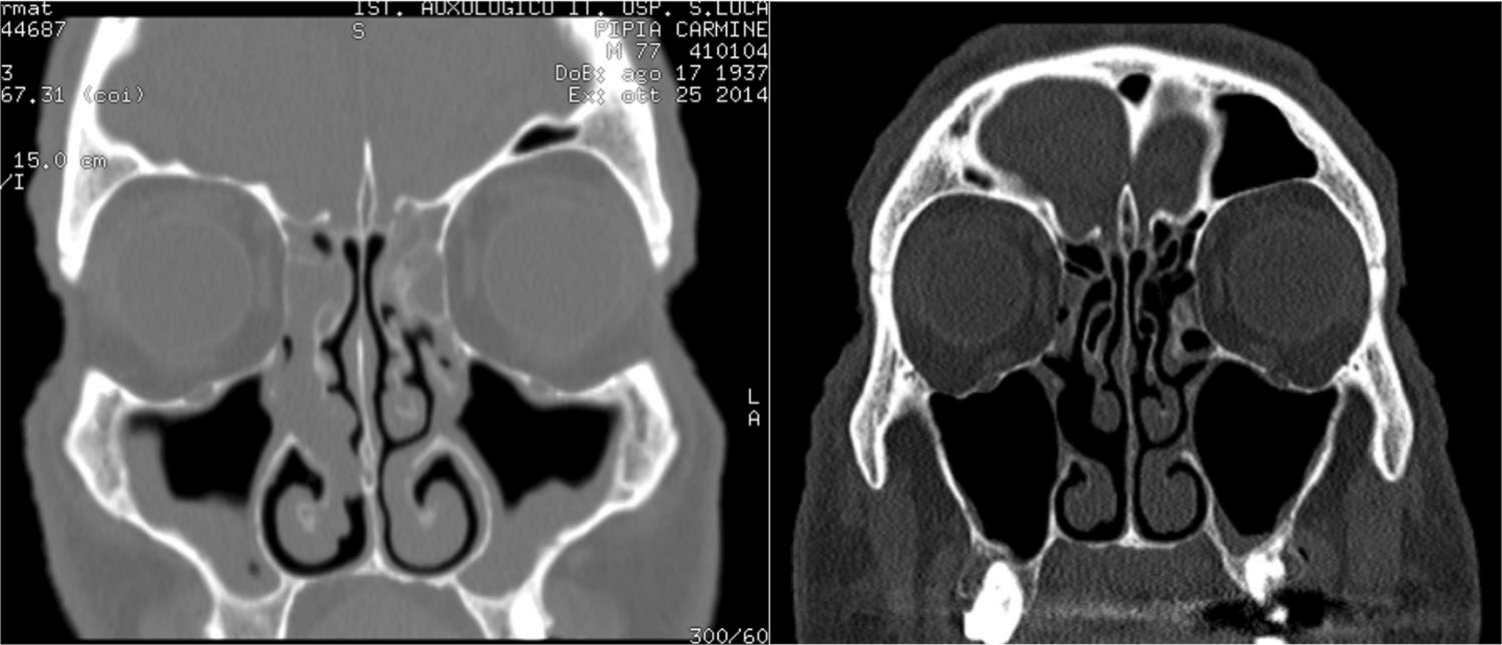
Example of a CT scan’s patient at T0 (on the left side) and at T4 (on the right side) of dupilumab treatment

Median value of peripheral eosinophils, total IgE levels, SNOT22, ACT, NPS, rhinomanometry and sniffing test measured at each timepoint evaluations are reported in Table 2. A Wilcoxon signed-rank test has shown a statistically significant improvement of SNOT22, olfactometry at Sniffin' sticks test (part identification) and NPS scores in each pairwise comparison (see Table 3) (Fig. 1). We did not observe a worsening of pneumological outcomes in terms of ACT score (Table 3). Eosinophils count have shown a slight increase up to 6 months (T2), then decreasing up to the end of the follow up period (Table 2).

**Table 2:** Mean values and IQR (interquartile range) in brackets of peripheral eosinophilia (10^9^/L), total IgE (kU/L), SNOT22, ACT, NPS, rhinomanometry and sniffing test resulted at T0, T1 and T2 evaluations

	T0	T1	T2	T3	T4
Eosinophils	0.40 (0.11–0.60)	0.90 (0.30–1.04)	0.85 (0.50–2.10)	0.50 (0.35–0.85)	0.48 (0.20–0.72)
blood IgE	312 (156–361.5)	50 (26.9–206)	32.5 (26.25–58.65)	33 (11.5–62.8)	21 (11.4–31)
SNOT22	56 (49–69)	26 (21–33)	15 (10–28)	13 (11–18)	12 (2–18)
ACT	20 (17–24)	24 (21–25)	24 (21–25)	24 (23–25)	24 (22–24)
NPS	5 (3–6)	1 (0–2)	0 (0–1)	0 (0–0)	0 (0–1.25)
Rhinomanometry—right nostril	0.76 (0.58–1.42)	0.56 (0.42–0.99)	0.66 (0.36–1.05)		
Rhinomanometry—left nostril	1.15 (0.76–1.33)	0.45 (0.23–0.70)	0.83 (0.62–1.44)		
Sniffin’ sticks test	3 (2–6)	11 (9–12)	12 (9.75–13)	12 (9.5–13)	12 (9.5–14)

**Table 3:** Statistical analysis

	T0–T1	T0–T2	T0–T3	T0–T4
Eosinophils	0.004	0.02	0.17	0.678
IgE	0.018	0.012	0.028	0.043
SNOT22	< 0.001	< 0.001	< 0.001	0.005
ACT	0.041	0.014	0.018	0.038
NPS	< 0.001	< 0.001	< 0.001	0.005
Rhinom dx	0.058	0.327	–	–
Rhinom sin	0.017	1	–	–
Olfactometry	< 0.001	< 0.001	< 0.001	0.013

*Rhinom* rhinomanometry

Three patients out of 20 have dropped out of the study due to adverse effects, poor asthma control or clinical status not depending on biological therapy.

- One patient was switched back to previous biological therapy due to poor control of her asthma after six months from switching to dupilumab (at T2 evaluation ACT = 11) which required hospitalization. Further examination showed that sinonasal symptoms have remained stable with SNOT22 score of 54 3-months later, while endoscopy showed significant reduction of NPS.
- One patient has discontinued the study at 12 months (T3) due to the late onset of psoriasis. His absolute eosinophil count has been recorded at approximately 2.7 × 10^9^/L, and the total IgE value corresponded to 25 kU/L. Despite this, sinonasal symptoms have been therapeutically controlled, resulting in NPS score of 2 and SNOT22 of 14 at T3 evaluation, while asthma symptoms remained moderately controlled with ACT of 21. The patient has then been reassigned to the previous biological therapy with Mepolizumab. Anyway, it has to be noted that despite an initial regression of psoriatic lesions after the drop-out, cutaneous signs started to reappear after 1 month from the reintegration of Mepolizumab, even with level of eosinophils of 0.28 × 10^9^/L.
- One patient has been hospitalized due to thrombocytopenia (platelet count of 19,000 per μL), due to a subsequent diagnosis of medullary aplasia. However, control of sino-nasal symptoms has been adequate (SNOT-22 at T5 = 20; NPS = 0), and bronchial asthma was well managed (ACT = 22). At one year after the drop-out, the patient still registers stable parameters: SNOT22 28 and NPS 0.

## Discussion

The recent advent of monoclonal antibodies for the pharmacological management of type2-driven CRSwNP has led to interesting results. Indeed, a systematic review of 2020 reported response rates for CRSwNP with comorbid asthma undergoing biologic treatment of between 50 and 70%, showing how response to biologics may not be all encompassing and that not all patients equally benefit from them [4].

Clinicians are still investigating on which biologic could fit best for each patient, as response is still heterogenous if considering all approved biologics for asthma and CRSwNP. An hypothesis is to indirectly compare the effects on CRSwNP in terms of selected end points [11]. Accordingly, the last few years have seen an increase in systematic reviews and meta-analysis comparing the different monoclonal antibodies. Data analysis reveals dupilumab to be the most favorable in terms of efficacy and safety profile, considering NPS, SNOT-22, UPSIT, and NCS scores [16–18].

Nevertheless, the subtyping of the CRSwNP population to choose which biologic could fit best is still poorly investigated. In this respect, a multicentric observational study by De Corso et al. [19] focused on 648 patients with CRSwNP treated with dupilumab and especially on their comorbidities and clinical history. NPS and SNOT-22 scores had a substantial decrease in patients undergoing dupilumab treatment, with a steeper decrease in patients who had prior surgeries and had comorbid asthma.

Other recent systematic reviews and network meta-analysis [20], compared the efficacy of monoclonal antibodies for treating CRSwNP and they highlighted limitations in comparing data of clinical trials. The heterogeneity in study populations and its enrollment requirements present noteworthy constraints and the differences between symptoms measurements, rate of previous surgeries and the use of OCS, entail important divergences in study design and therefore limit the comparability of its outcomes [20, 21].

It is, therefore, of the utmost importance, to comprehensively determine the indications and relative efficacy of these agents, to provide homogeneous real-life studies to include comparable patient populations and standardize outcome measures. This supports the importance of evaluating patients individually, taking comorbidities and patient’s profiles into consideration.

Otten et al. [22] describe the need of switching to other biologics in 94 patients with CRSwNP and asthma who were not adequately controlled in terms of upper and/or lower airways symptoms. While the authors try to provide an algorithm to help choosing the best therapeutic management in cases of non-responders, the study shows only general explanations of poor or good response to treatment. It does not highlight specific endpoints to compare responses to each biologic, nor evaluate clinical evolution at established time points, reporting just NPS outcome at 6 months after switching biologics of two patients.

Regarding our results, they confirmed the evidence found in literature on improving CRSwNP outcomes by dupilumab, however, also in those patients not responding to omalizumab or mepolizumab/benralizumab. In particular, we registered a significant improvement in all evaluated parameters at each time point: SNOT22, NPS, rhinomanometry, Sniffin’stick test and total IgE (Table 2).

Odor perception has considerably increased in our series, shifting from a median value of Sniffin’ stick 3/16 at T0 to 12/16 at T4 (Table 2). Further olfactory measurement such as discrimination and threshold testing would be necessary to evaluate olfaction and olfactory gain under dupilumab, but our findings strengthen the literature’s evidence of dupilumab as first choice in terms of smell improvement [16, 18, 23].

Total IgE has shown a progressive decrease, while peripheral eosinophils had a steady increase up to 6 months, followed by a decrease registered up to 18 months of follow-up (Table 2). It is worth noting that the majority of these patients began the dupilumab treatment with only a moderate level of blood eosinophils, as these were in part responsive to the previous monoclonal therapy. Literature confirms our findings postulating the reduction of eosinophil recruitment from tissues during dupilumab treatment, thus a subsequent increase of blood eosinophils with a peak at around 16–20 weeks from the beginning of therapy, followed by a slow decrease [22, 24]. Of note is the longer period of increase of our patients before reaching a steady state. Literature debates whether all cases of hypereosinophilia correlate with any onset of hypereosinophilic syndrome or other clinical manifestation [24]. Notably, in our analysis only one patient out of 12 with blood hypereosinophilia during treatment, has shown pruritus and psoriasis and had to stop treatment, which we postulated may be related to the hematological finding.

Pulmonary function questionnaires showed stable therapeutic control of severe asthma. The only exception was one patient which had to stop treatment after T2 evaluation due to an asthma exacerbation. Hence, this suggests how the control of CRSwNP by dupilumab does not directly imply a concomitant good control of severe asthma in the totality of cases. The risk of loss of asthma control, although very low as shown by our data, should be discussed with the patient before any shift. Once again, it highlights the need of finding means for subtyping the type-2 population to identify these non-responders precociously [25].

Even though our case series shows only a very limited number of non-responders/adverse effects of dupilumab, we may assume that efficacy can at times differ in terms of CRSwNP and simultaneous comorbid asthma control. We experienced a higher rate of adverse effects than in first-line treatment with dupilumab; also Otten et al. evidenced in their analysis about managing comorbid patients not responding to the first-choice biologic, that second-line dupilumab seems to be the biologic with most adverse events, mainly hyper-eosinophilia which can be difficult to treat in a minority of patients. To overcome this issue, they propose to switch again to or combine with an anti-IL5 treatment which lowers eosinophils’ blood count [22]. Notably, another study testing the effectiveness of modifying the biological therapy from omalizumab to dupilumab in 23 patients reported an interesting rate of 4.3% keratocongiuntivitis sicca, which lead to termination of therapy in one patient [26]. In literature there is no evidence of congiuntivitis in first-line treatment with dupilumab in CRSwNP, so it is another confirm that probably switching from another biologic may trigger unexplored cross-reactions [27].

It was very interesting to experience one patient developing thrombocytopenia and thus medullary aplasia. To our knowledge, no cases of thrombocytopenia or related medullary aplasia are reported in literature, neither for the use of dupilumab for other clinical indications [28, 29]. A case report talks about an immune thrombocytopenic purpura in a patient with atopic dermatitis treated with dupilumab: the correlation was explained with a combination of nasopharyngitis, cefuroxime intake and reversion of a Th1/Th2 imbalance by dupilumab as possible trigger factors for immune thrombocytopenic purpura [30]. Anyway, our patient did not get an immune disorder, but rather a medullary disfunction which did not seem related to dupilumab intake.

Unfortunately, studies with monoclonal antibodies directed at type 2 endotypes have not found reliable biomarkers to predict response to treatment [12]. At the moment the combination of phenotype, symptoms, response to treatment and markers like eosinophils and IgE either in blood or tissue lead to the best approximation of the expected response to treatment. Consequently, in real-life, once we have ascertained the presence of a type 2 inflammation, we may only observe subsets of patients to be either responder or non-responder.

In our study, we observed a low incidence of adverse effects in patients who were treated with dupilumab, according to literature [27, 31]. We experienced one asthma exacerbation and one worsening of psoriasis accompanied by increased pruritus and hypereosinophilia in one patient which led to discontinuation of dupilumab. It has to be noted, however, that the latter one had a recurrence of psoriatic lesions even after the return to the previous biological therapy with Mepolizumab and at a normal level of blood eosinophils, suggesting how the pathophysiology underlying these cutaneous lesions may not be only referred to eosinophils’ count.

Based on the SUCRA (surface under the cumulative ranking) values for safety (AEs), dupilumab reported slightly higher frequencies of cough, bronchitis, arthralgia, accidental overdose, and injection-site reactions, compared to other biologics [17]. Notwithstanding, a review of the literature by Nitro et al. (2022), highlights that different phenotypes of type 2 inflammation patterns (asthma versus EOS versus CRSwNP versus AD) exhibit distinct side effects, as for e.g. the above mentioned reactions for CRSwNP patients with no reports of symptomatic eosinophilia or ophthalmological or dermatological manifestations except for one case of dermatitis [27].

This study sheds light on an important limit of current use of these new agents: each biologic is directed towards a specific target of type-2 inflammation process, and the phenotype correspondence is yet to be discovered. Indeed, only a subgroup of all patients in biologic treatment for asthma with comorbid CRSwNP, did not show a satisfying response in terms of CRSwNP and were therefore included in this study. This introduces its most important limitation as a selection bias: we did not analyze all the patients with an already good response both for severe asthma and CRSwNP with Benralizumab, Omalizumab and Mepolizumab. Therefore, we are inevitably unable to define here whether a biologic is superior in terms of CRSwNP clinical control.

Literature still lacks real life comparative studies: it would be extremely valuable to be able to match each biologic with the phenotype-endotype of patients which could benefit the most from a specific molecule, and hence to customize biological therapy for specific subsets of CRSwNP/comorbid CRSwNP.

Moreover, as we evaluated only a small case series, we are not able to provide reliable and replicable data regarding the wider population. A 18 months-follow-up limits the potential observation of long-term adverse effects, complications or efficacy regarding both the control of asthma and CRSwNP [21].

## Conclusions

Response to treatment with monoclonal antibodies seems to be heterogeneous and could be based on specific clinical profiles. In our study, dupilumab, appears to be promising in cases that did not reach satisfying clinical control of comorbid CRSwNP with other biologic therapies. Future real-life success will rely on the availability of biomarker-based endotyping and responder analyses, to allow for matching of each patient with the appropriate biologic, thereby optimizing treatment strategies.

## Data Availability

The data that support the findings of this study are available from the corresponding author upon request.
